# Clustering on Magnesium Surfaces – Formation and Diffusion Energies

**DOI:** 10.1038/s41598-017-05366-1

**Published:** 2017-07-12

**Authors:** Haijian Chu, Hanchen Huang, Jian Wang

**Affiliations:** 1 Department of Mechanics, Shanghai University, Shanghai, 200444 China; 2 Shanghai Institute of Applied Mathematics and Mechanics, Shanghai University, Shanghai, 200444 China; 3 Department of Mechanical and Industrial Engineering, Northeastern University, Boston, MA 02115 USA; 4 Department of Mechanical and Materials Engineering, University of Nebraska-Lincoln, Lincoln, NE 68588 USA

## Abstract

The formation and diffusion energies of atomic clusters on Mg surfaces determine the surface roughness and formation of faulted structure, which in turn affect the mechanical deformation of Mg. This paper reports first principles density function theory (DFT) based quantum mechanics calculation results of atomic clustering on the low energy surfaces {0001} and $\{\bar{1}011\}$. In parallel, molecular statics calculations serve to test the validity of two interatomic potentials and to extend the scope of the DFT studies. On a {0001} surface, a compact cluster consisting of few than three atoms energetically prefers a face-centered-cubic stacking, to serve as a nucleus of stacking fault. On a $\{\bar{1}011\}$, clusters of any size always prefer hexagonal-close-packed stacking. Adatom diffusion on surface $\{\bar{1}011\}$ is high anisotropic while isotropic on surface (0001). Three-dimensional Ehrlich–Schwoebel barriers converge as the step height is three atomic layers or thicker. Adatom diffusion along steps is via hopping mechanism, and that down steps is via exchange mechanism.

## Introduction

Hexagonal metals in a bulk form are widely used in nuclear engineering^1^ and transportation industry (magnesium and magnesium alloys are the lightest structural materials)^2^. Mg and Mg alloys exhibit low strength and poor deformability because of the easy twinning. Nanometer-sized hexagonal materials show significantly high strength, 5 to 10 times of the bulk strength^3, 4^. However, deformation twin quickly propagates across the sample once it occurs, corresponding to the burst of plastic deformation^5^. Planar defects such as twin boundaries, stacking faults, and interphase interfaces can impede the motion and propagation of dislocations and twins, thus strengthening materials^6–10^. To realize planar defects in Mg and Mg alloys, it is essential to understand atomic clustering processes during material processing (synthesizing and aging). For example, alloying with appropriate solutes alloying is very promising in tailoring mechanical properties of Mg alloys^11^. Shin and Wolverton^12^ calculated solute–vacancy binding in Mg alloys using principles density function theory, and Ganeshan *et al*. performed first principles density function theory (DFT) calculations of self-diffusion in hcp Mg^13^. Understanding of solute clustering processes in Mg alloys has provided insight in modifying the dispersion and morphology of precipitates.

Apart from clustering processes in the bulk Mg, planar defects in nanostructured Mg and Mg alloys can also be introduced during synthesizing, such as sputtering via physical vapor deposition techniques^14–20^. Atomic clustering on surfaces during a growth process of thin films or nanostructures dictates the structure and their mechanical properties. The clustering processes depend on formation and diffusion energies of adatom or atomic clusters. If the formation energy of an adatom or an atomic cluster in a fault stacking is lower than in a normal stacking, stacking faults may form. In addition, the diffusion kinetic barriers of adatom or atomic clusters dictate whether a cluster can easily move into a fault or normal packing configuration. For example, on (111) surface of a face centered cubic (fcc) crystal, an adatom or an atomic cluster can take two stacking sites, either fcc sites or hexagonal closed pack (hcp) sites. As a consequence, the new layer can grow into either normal fcc structure or a stacking fault structure which further leads to twin structure^21–24^. The stability of a fault layer is dependent on both the formation energy and especially corresponding diffusion energy of the cluster. Atomic clustering processes have been intensively studied for materials with cubic structures using first principles density function theory^25–29^, atomistic simulations with empirical interatomic potentials^30–35^, and experiments coupling with theoretical interpretation^36, 37^. A few studies were done for hexagonal metals. Using molecular statics (MS) method with empirical interatomic potential, Johansen *et al*. calculated diffusion and formation energies of adatoms and vacancies on five surfaces of magnesium, {0001}, $\{1\bar{1}01\}$, $\{11\bar{2}0\}$, $\{1\bar{1}00\}$A and $\{1\bar{1}00\}$B^38^, and successfully predicted surface orientations in Mg nanoblades synthesized by physical vapor deposition and dominant side surfaces $\{1\bar{1}01\}$ of Mg nanoblades^39^. Wu *et al*. recently created faceted nanopores in magnesium by aligning the electron beam (e-beam) along the [0001] direction^40, 41^. The formation of hexagonal prism-shaped and hourglass-shaped three-dimensional morphologies^40^ is attributed to adatom diffusion kinetics and surface energegetics. However, atomic clustering processes, such as formation and diffusion energies of clusters and three-dimensional Ehrlich–Schwoebel barriers associated with adatom diffusion along and down steps^42^, have not been studied.

In this work, we investigated formation and diffusion energies of clusters and three-dimensional Ehrlich–Schwoebel barriers in Mg. Unlike for cubic metal, hexagonal metal is not so well represented by empirical interatomic potentials. We choose to use DFT based quantum mechanics calculations as the reference in this study. By repeating the calculations using empirical interatomic potentials based MS calculations, we verify the reliability of such potentials, and then choose a reliable potential to extend the DFT studies.

## Results

### Comparison between DFT and empirical potentials

**Figure 1:**
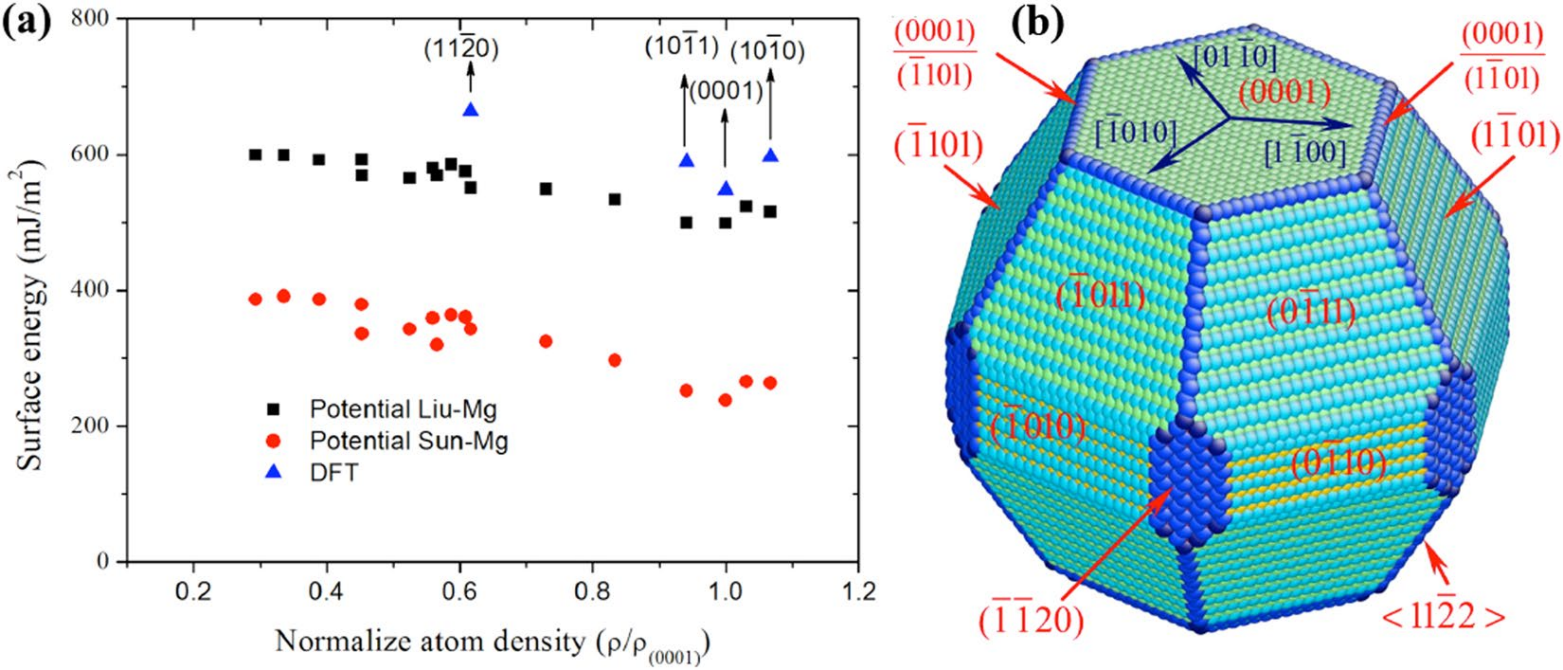
(**a**) The formation energies as a function of atomic areal density. Four compact surfaces were calculated using DFT because of the size limit of DFT calculations. (**b**) The Wulff structure shows two major surfaces (0001) and $\{\bar{1}011\}$ and two minor surfaces $\{\bar{1}010\}$ and $\{\bar{1}\bar{1}10\}$.

Atomistic simulations have been demonstrated to be powerful tools in exploring energetics and kinetics of surface defects. However, DFT calculation is limited by simulation size and time, molecular statics/dynamics (MS/MD) simulations are capable for large scale and long time simulations. The reliability of MS/MD simulations is related to whether the empirical interatomic potential can re-produce energetics and kinetics of the studied events. Two existing, widely used empirical interatomic potentials are compared with DFT calculations. The models and corresponding computation details are described in Supplementary S1. The formation energy of 16 surfaces with respect to atomic areal density of surfaces is plotted in Fig. 1a and summarized in Table 1. The horizontal axis represents the normalized atomic areal density of the double-terminated plane that is normalized by the density of surface (0001). Surface energy decreases with the increase of atomic areal density of surfaces. Surface energies obtained with Liu-Mg potential^43^ are much closer to the DFT results with the relative error in the range of 9.0~17.0%, while, Sun-Mg potential^44^ with a much bigger relative error in the range of 48.3~57.2%. We further examined the capability of Liu-Mg potential for surface vacancy. For a vacancy within surface (0001), DFT calculation shows the formation energy of 0.53 eV and MS calculation with Liu-Mg potential gives 0.43 eV. A relative error is 18.8%. For a vacancy within surface $\{\bar{1}011\}$, DFT calculation shows the formation energy of 0.41 eV and MS calculation with Liu-Mg potential gives 0.38 eV. A relative error is 6.2%. In addition, we also compared the kinetic barriers associated with surface diffusion of adatom, dimer, and trimmer. The details will be discussed in following sections. These results suggest the reliability of Liu-Mg potentials in performing large-scale molecular dynamics simulations. With these surface energies obtained from DFT and Liu-Mg potential calculations, we construct the Wulff structure in Fig. 1b that shows two major surfaces (0001) and $\{\bar{1}011\}$ and two minor surfaces $\{\bar{1}010\}$ and $\{\bar{1}\bar{1}20\}$. In what follows, we studied kinetics and energies of surface clusters and surface steps on surfaces (0001) and $\{\bar{1}011\}$ using DFT and MS simulations with Liu-Mg potential.

**Table 1:** Surface formation energy (mJ/m^2^). Areal density denotes the normalized atomic areal density *ρ*/*ρ*_{0001}_.

$$\{\bar{{\bf{1}}}{\bf{01}}{\bf{n}}\}$$								
*n*	(0001)	4	3	2	1.5	1	0.5	0
Areal density	1.00	0.452	0.566	0.730	0.833	0.941	1.031	1.067
DFT	548.3					589.3		596.9
Liu-Mg	499.0	569.5	569.7	549.6	534.0	499.7	523.8	515.5
Sun-Mg	238.0	335.9	319.8	324.8	297.0	252.2	264.9	263.1
$$\{\bar{{\bf{1}}}\bar{{\bf{1}}}{\bf{2}}{\bf{n}}\}$$								
*n*	5	4	3	2	1.5	1	0.5	0
Areal density	0.335	0.388	0.452	0.454	0.580	0.587	0.608	0.616
DFT								663.9
Liu-Mg	599.2	591.8	592.4	565.5	580.4	585.5	575.4	551.2
Sun-Mg	391.1	386.5	379.1	342.5	358.6	361.6	360.4	343.1

### Clusters on surface {0001}

**Figure 2:**
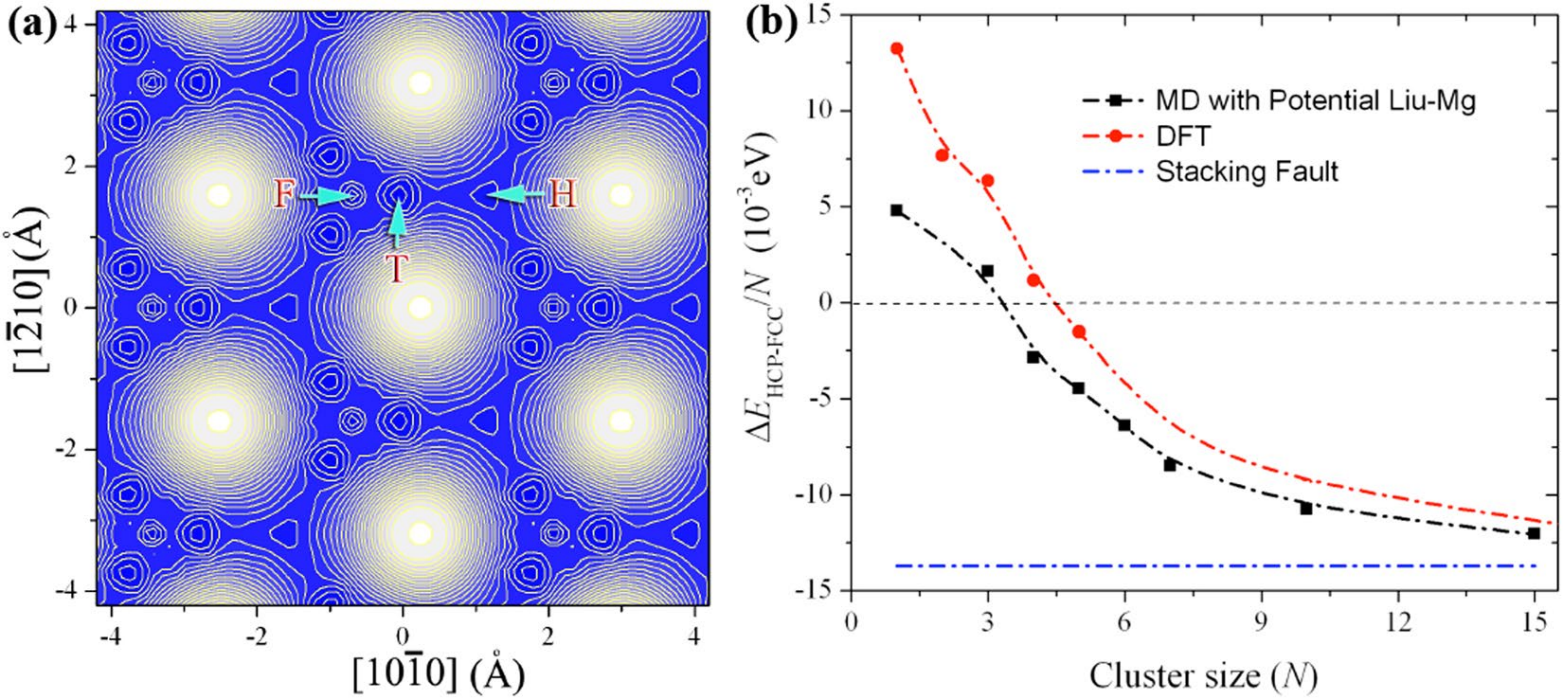
(**a**) The surface potential landscape in association with diffusion of an adatom on surface (0001), revealing three local stabilized sites, the fcc site (F-site), the hcp site (H-site) and the tetrahedral site (T-site) in a fcc structure. (**b**) The variation of the energy difference between the fcc and hcp structures with the cluster size. Δ*E*_*HCP*−*FCC*_/*N* represents the average energy difference between the fcc and hcp structures of a cluster on the surface. The blue dashed line indicates the energy difference corresponding to the stacking fault in hcp structure.

Fig. 2a shows the surface potential landscapes of adatom diffusion that are obtained using DFT calculations and MS calculations with Liu-Mg potential. The simulation models are described in Supplementary S2. Both reveal three local stabilized atomic sites, the fcc site (F-site), the hcp site (H-site) and the tetrahedral site (T-site) in a hcp structure. DFT calculations show that F-site has the lowest energy, while H-site has the highest energy with the relative difference of 0.013 eV to the F-site, and T-site is in-between them with the relative difference of 0.003 eV to the F-site. An adatom diffuses on surface from H-site to T-site then to F-site, experiencing the barriers 0.005 eV and 0.015 eV; or from F-site to T-site then to H-site, experiencing the barriers 0.018 eV and 0.015 eV. MS calculations with the Liu-Mg potential show that T-site has the lowest energy, F-site has the highest energy with the relative difference of 0.028 eV to the T-site, and H-site is in-between them with the relative difference of 0.023 eV to the T-site. An adatom diffuses on the surface from T-site to F-site with a kinetic barrier of 0.030 eV and from T-site to H-site with the kinetic barrier of 0.026 eV; or from F-site to T-site with the kinetic barrier of 0.002 eV and from H-site to T-site with the kinetic barrier of 0.003 eV. Both DFT and MS calculations show that the H-site has the highest energy, while there is different order between the two sites F-site and T-site. This indicates that an adatom is not energetically located at the H-site. Small cluster on the surface (0001) may prefer to form FCC structure not the HCP structure. In addition, the energy barriers and the energy difference among the three sites are really small (<0.03 eV), implying that the diffusion is very efficient among these sites at room temperature. Thus Liu-Mg potential will not cause substantial error for simulating crystal growth associated with adatom migration on (0001) surface at room temperature.

There must be a critical size of clusters that corresponds to the transition from fcc-preferred structure to hcp-preferred structure. We tested this hypothesis by comparing formation energy of surface clusters. For example, a dimmer could have more than 9 different configurations corresponding to each atom occupying one of the three low sites (F, H and T sites). Corresponding to the crystal growth, we only calculated the formation energy of a cluster when they all locate at fcc sites and hcp sites. Even so, a cluster still can adopt multiple configurations. For example, a three-atom cluster has 4 configurations (S.3 and Supplementary Fig. S3). Figure 2b shows the energy difference between fcc and hcp sites of clusters with respect to the cluster size. The critical size *N* of clusters is found to be 3 for the empirical potential and 4 for DFT. We also calculated the energy barrier in association with the transition of 3-atom, 4-atom, and 5-atom clusters between fcc and hcp sites (Supplementary S4) using nudge-elastic-band method^45^. For each sized clusters, we tested several possible diffusion paths, see the details in Supplementary Figures S4, S5 and S6. The energy barrier is 0.028 eV, 0.035 eV, and 0.038 eV from fcc sites to hcp sites, and 0.037 eV, 0.047 eV, 0.061 eV from hcp sites to fcc sites, for the transition of 3-atom, 4-atom, and 5-atom clusters, respectively.

### Clusters on surface ${\boldsymbol{\{}}\bar{{\bf{1}}}{\bf{011}}{\boldsymbol{\}}}$

**Figure 3:**
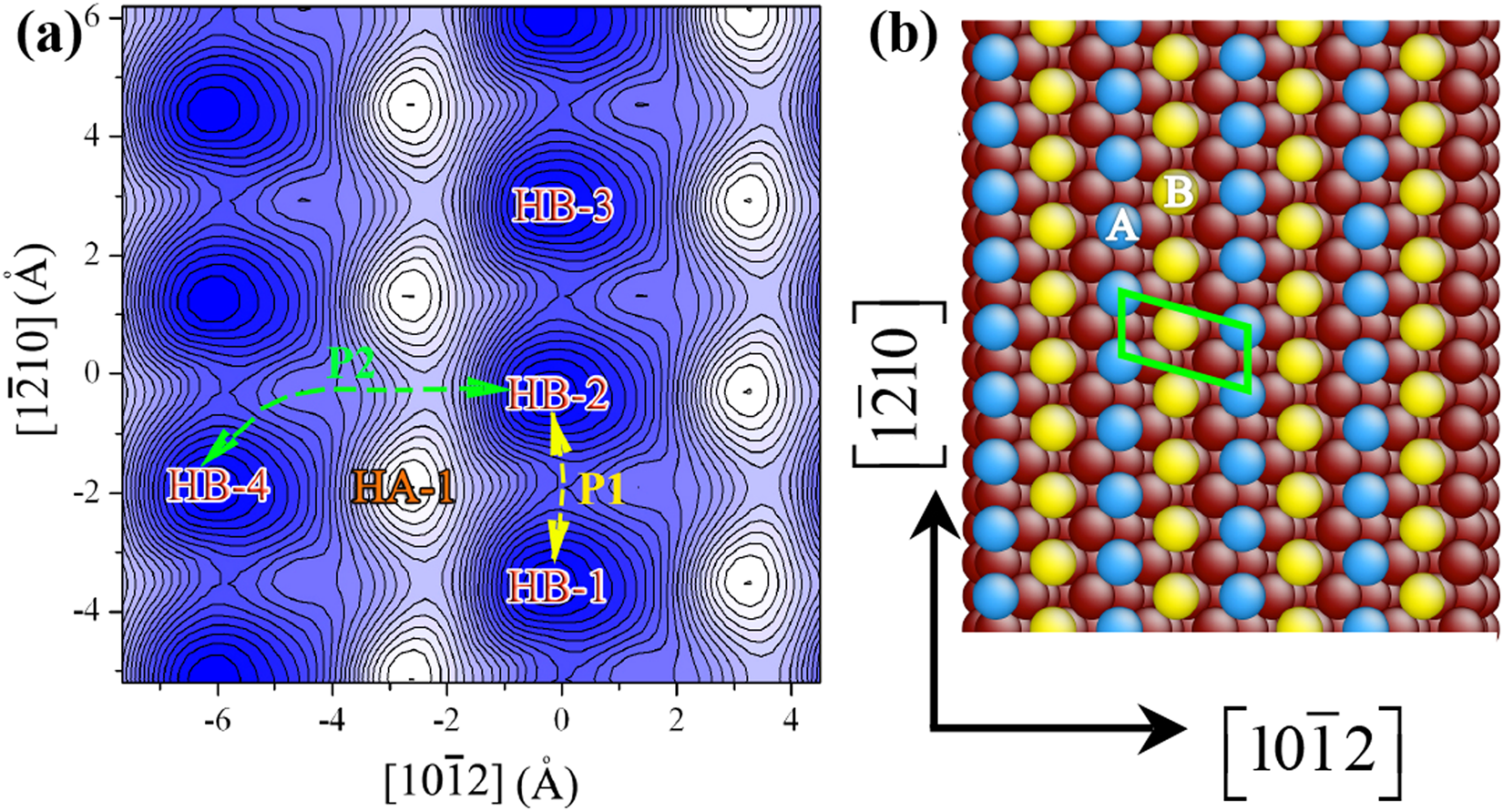
(**a**) Atomic structure of surface $\{\bar{1}011\}$. (**b**) The potential landscape in association with the diffusion of an adatom on ($\bar{1}011$) surface. HA and HB indicate the lattice sites.

Surface $\{\bar{1}011\}$ is a rumpled crystallographic plane, which could be terminated by plane A or plane B or a combined A-B plane, indicated in Fig. S1. From the energetic point of view, the surface termination with A and B planes together would be preferred, but it is not clear how the kinetics drives the terminated plane. Figure 3 shows surface potential landscapes for adatom diffusion that are obtained by the DFT and MS with Liu-Mg potential (Supplementary S5). Both reveal one stabilized site corresponding to the hcp site on plane B, referred to as HB and one metastable hcp site corresponding to a hcp site on plane A, referred to as HA, as indicated in Fig. 3. The HA-site has the highest formation energy 0.49 eV, indicating that an adatom is energetically located at the HB-site during surface growth. Regarding kinetics processes, adatom diffusion on the surface will take two pathways from HB to HB along $\langle 1\bar{2}10\rangle$ (referred 25%), and along P2 across $\langle 1\bar{2}10\rangle$ with the energy barrier of 0.37 eV (DFT) and 0.31 eV (MS) (a relative error of 16%). Both reveal the minimum energy path along the compact direction $\langle 11\bar{2}0\rangle$ and the same diffusion trend between the paths P1 and P2, implying that Liu-Mg potential is reliable for simulating the crystal growth in association with adatom motion on surface ($\bar{1}011$).

We further studied the formation energy of clusters with respect to the cluster configuration. A dimer may take three different configurations, (a) on HB-1 and HB-2 along $[1\bar{2}10]$, (b) one on HB-1 and the other on HA-1, and (c) one on HB-1 and the other on HB-4 across a HA site. Configuration (a) has the lowest formation energy. Configuration (b) has the formation energy, 0.24 eV higher than configuration (a), and configuration (c) has the formation energy, 0.27 eV higher than configuration (a). The results imply that the growth of surface ($\bar{1}011$) is energetically preferred along $\langle 1\bar{2}10\rangle$, and the metastable site HA becomes energetically stable once its neighboring HB site is occupied. A trimmer has three configurations, (a) three HB sites along $\langle 1\bar{2}10\rangle$ (including HB-1, HB-2, and HB-3 sties), (b) two HB sites (HB-1 and HB-2) and one HA site (HA-1), and three HB sites across a HA site (including HB-1, HB-2, and HB-4), MS calculations show that configuration (a) has the lowest formation energy, and configurations (b) and (s) have the formation energy, 0.16 eV and 0.22 eV higher than configuration (a). These results imply that surface $\{\bar{1}011\}$ is terminated by both *A* and *B* planes, forming a rumpled crystallographic plane, and the growth is preferred along the compact direction $\langle 1\bar{2}10\rangle$.

### Steps on surface (0001)

**Figure 4:**
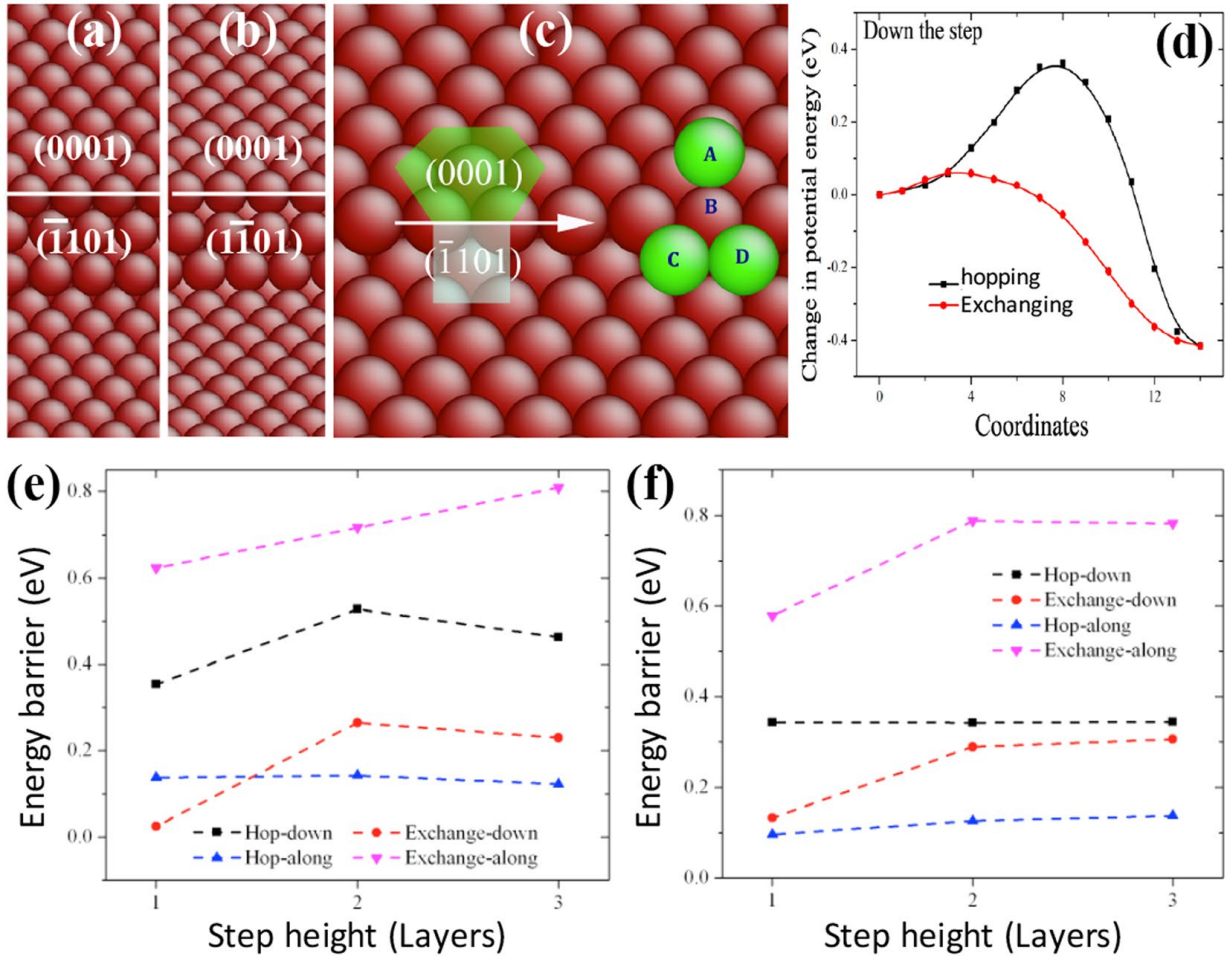
(**a**) Diffusion paths for adatom in the left step corresponding to the ledge between (0001) and ($\bar{1}101$), (**b**) Minimum energy path of adatom diffusion along the left step by exchanging and hopping mechanisms, (**c**) Diffusion paths for adatom down the one-layer step between (0001) and ($\bar{1}011$) by hopping (A → C) and exchanging (A → B → C), (**d**) The change in potential energy corresponding to the two diffusion paths described in (**c**), (**e**) Variation of kinetic energy barriers as a function of the step height associated with an adatom diffusion down the left step by exchanging and hopping mechanisms, and (**f**) Variation of kinetic energy barriers as a function of the step height associated with an adatom diffusion down the right step by exchanging and hopping mechanisms.

Ehrlich–Schwoebel barriers have been demonstrated to be step height dependence^42^. According to the crystallography of the Wullf structure, the six $\langle 1\bar{2}10\rangle$ -oriented ledges are related to adatom diffusion across facets (0001)-($\bar{1}101$) and (0001)-($1\bar{1}\,01$), as shown in Fig. 4a,b. The reliability of Liu-Mg potential is tested for adatom diffusion along the $\langle 1\bar{2}10\rangle$ -oriented step. The detail is described in Supplementary S6. The energy barrier is 0.124 eV (MS) and 0.139 eV (DFT), with a relative error of 10.8%, which is reasonably accepted for atomistic simulations with empirical potential. Here we performed a systematic study of adatom diffusion down and along steps with different heights. For each step, an adatom can diffuse along and down the step by exchanging and hopping mechanisms.

Adatom diffusion along and down steps with the facet ($\bar{1}101$) is summarized in Supplementary S7. Figures S8, S9 and S10 show atomic structures of one-layer step, two-layer step, and three-layer step, as well as diffusion paths and the corresponding energy profile associated with adatom diffusion. Figure 4c shows diffusion paths for adatom down the one-layer step between (0001) and ($\bar{1}101$) by hopping (A → C) and exchanging (A → B → C). Figure 4d shows the change in potential energy corresponding to the two diffusion paths. The results are summarized in Fig. 4e. Adatom diffusion down the step by exchanging will experience a low barrier, 0.063 eV for one-layer step, 0.235 eV for the 2-layer step, and 0.234 eV for the 3-layer step; but adatom diffusion along the step by hopping will experience a low barrier, 0.139 eV for all steps.

Adatom diffusion along and down steps with the facet ($\bar{1}101$) is summarized in Supplementary S8. Figures S11, S12 and S13 show atomic structures of one-layer step, two-layer step, and three-layer step, as well as diffusion paths and the corresponding energy profile associated with adatom diffusion. The results are summarized in Fig. 4f. Adatom diffusion down the step by exchanging will experience a low barrier, 0.186 eV for the one-layer step, 0.235 eV for the 2-layer step, and 0.234 eV for the 3-layer step; but adatom diffusion along the step by hopping will experience a low barrier, 0.096 eV for all steps. Comparing the energy barriers for adatom diffusion between the two facets, we found that adatom diffusion down the step with the facet ($\bar{1}101$) has lower kinetic barrier than the facet ($\bar{1}101$), while adatom diffusion along the step with the facet ($\bar{1}101$) has the lower kinetic barrier than the facet ($\bar{1}101$), thus favoring the growth of (0001) toward the $[\bar{1}100]$, $[01\bar{1}0]$, and $[\bar{1}010]$ directions.

### Steps on surface ($\bar{{\bf{1}}}{\bf{101}}$)

**Figure 5:**
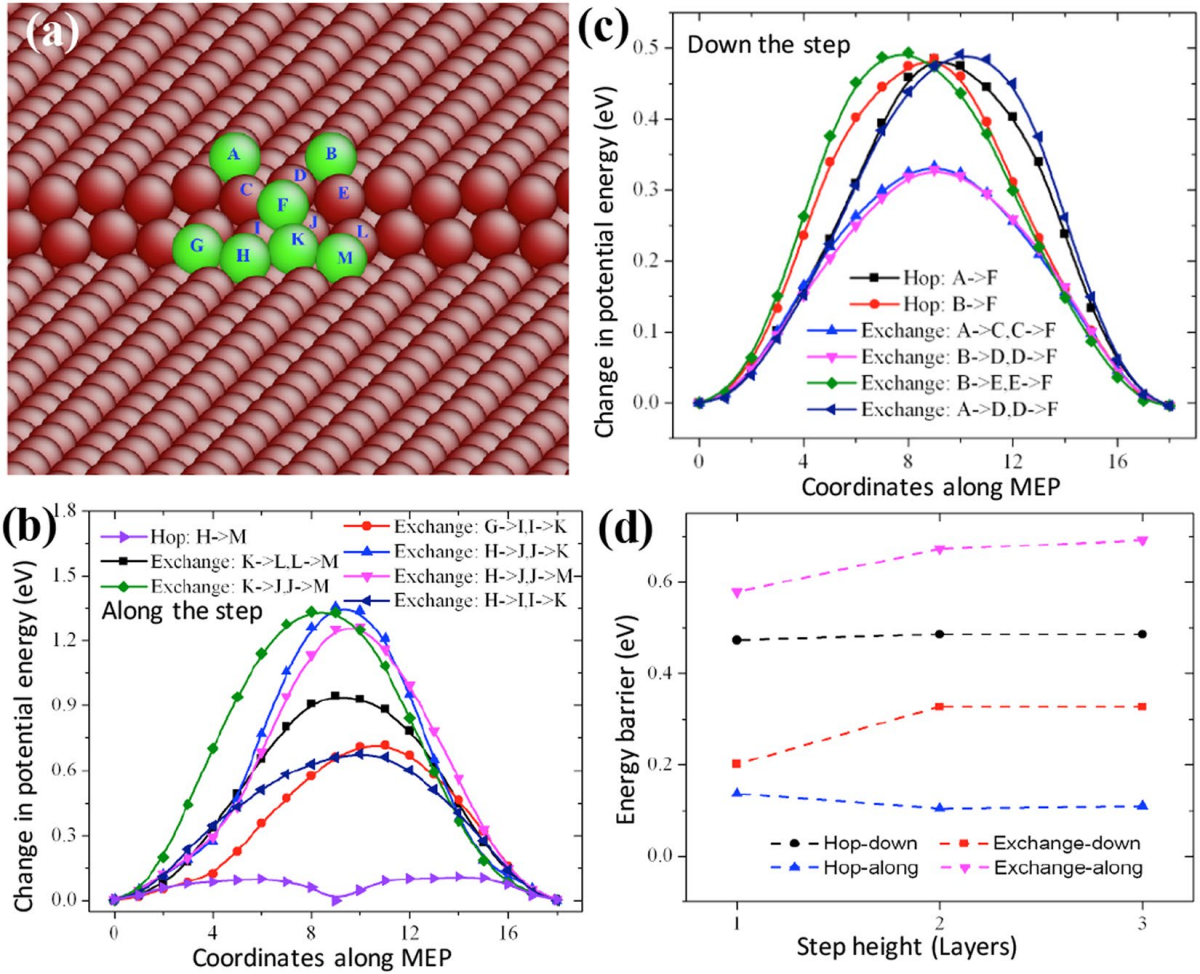
(**a**) Atomic structure of a 2-layer step on surface ($\bar{1}101$). (**b**) Change in potential energy with respect to different diffusion paths as an adatom diffuses along the step. (**c**) Change in potential energy with respect to different diffusion paths as an adatom diffuses down the step. (**d**) Variation of kinetic energy barriers as a function of the step height.

six $\langle \bar{1}\bar{1}23\rangle$ -oriented ledges are related to adatom diffusion across facets ($\bar{1}101$)-($1\bar{1}01$). Here we performed a systematic study of adatom diffusion down and along steps with different heights 1, 2, and 3. The details are summarized in Supplementary S9. For each step, an adatom can diffuse along and down the step by exchanging or hopping mechanisms. Figures S14, S15 and S16 show atomic structures of one-layer step, two-layer step, and three-layer step, as well as diffusion paths and the corresponding energy profile associated with adatom diffusion. Figure 5a shows diffusion paths for adatom down and along the 2-layer step. Figure 5b shows the change in potential energy corresponding to seven diffusion paths (one hopping case and six exchanging cases) as an adatom diffuses along the step. The minimum energy barrier is 0.110 eV via hopping mechanism. Figure 5c shows the change in potential energy corresponding to six diffusion paths (two hopping cases and four exchanging cases) as an adatom diffuses down the step. The minimum energy barrier is 0.332 eV via exchanging mechanism. The results are summarized in Fig. 5d. Adatom diffusion down the steps by exchanging experiences a low barrier, 0.203 eV for one-layer step, 0.332 eV for the 2-layer step, and 0.332 eV for the 3-layer step; but adatom diffusion along the step by hopping experiences a low barrier, 0.11 eV for the one-layer step, and 0.10 eV for the other steps.

## Discussion

Employing first principles density function theory, we studied the energies and kinetics of surfaces and surface clusters for magnesium with the hexagonal-close-packed structure. We also examined the same calculations using two existing empirical interatomic potentials Liu-Mg and Sun-Mg. It is worth mentioning that the existing empirical interatomic potential Liu-Mg is able to quantitatively and qualitatively reproduce the most important physics of surface defects. The relative errors of defects formation energy with respect to the DFT predictions are in the range of, 6~19% for vacancy in surfaces, ~10% for adatom diffusion, 9~17% for surfaces.

Surface formation energies of sixteen crystallographic planes were calculated using DFT and MS methods and compared. The results reveal that 1) the surface energy decreases with the increase of the atom density of the surface; 2) the close-packed plane (0001) has the lowest surface energy; 3) the Wulff structure of Mg has two major planes (0001) and $\{\bar{1}011\}$, and two minor plane $\{\bar{1}011\}$ and $\{\bar{1}\bar{1}20\}$; 4) surface energies obtained using Liu-Mg potential are much closer to the DFT results with the relative error in the range of 9~17%, but Sun-Mg potential gives a larger relative error in the range of 48~57%; (5) for a single vacancy, the formation energy in surface (0001) is 0.53 eV using DFT and 0.43 eV using Liu-Mg potential, the relative error is 18.8%; the formation energy in $\{\bar{1}011\}$ of 0.41 eV using DFT and 0.38 eV using Liu-Mg potential, a relative error is 6.2%. These results suggest that Liu-Mg potential is reliable in simulating crystal growth using molecular dynamics method.

On surface {0001}, we performed DFT and MS calculations for adatom, dimmer, trimmer, and clusters. The main results are (i) there are three local stabilized sites in association with an adatom, hcp site, fcc site and tetrahendral site. ii) A disk-like cluster composing of less than three atoms will be energetically stable on the fcc sites but unstable on the hcp sites, implying that a stacking fault structure is preferred during the growth for a small surface cluster. Correspondingly, iii) the kinetic barriers in association with the diffusion of surface clusters are calculated. The energy barrier in association with the transition of a four-atom cluster from fcc sites to hcp sites is 0.04 eV.

On surface $\{\bar{1}011\}$, adatoms and clusters prefer to sit on the hcp sites. Adatom diffusion from a HB site to the neighboring HB site experiences lower kinetic barrier of 0.27 eV along $\langle 1\bar{2}10\rangle$ while higher barrier of 0.37 eV across a HA site. The variation of the formation energy with respect to the cluster size and configuration implies that the growth on surface $\{\bar{1}011\}$ is terminated by both planes A and plane B together, growing a rumpled crystallographic plane.

Regarding adatom diffusion along and down a step on surface (0001), adatom diffusion along steps that are parallel to the compact direction $\langle 11\bar{2}0\rangle$ is accomplished via hopping mechanism with kinetic barrier of 0.10~0.14 eV, while adatom diffusion down such steps is accomplished via exchanging mechanisms with kinetic barrier of 0.24 eV. On surface $\{\bar{1}011\}$, adatom diffusion on surface $\{\bar{1}011\}$ is directional anisotropy, lower kinetic barrier of 0.31~0.37 eV as adatom diffusion along $\langle 11\bar{2}0\rangle$. Adatom diffusion down $\langle 11\bar{2}0\rangle$ steps on surface $\{\bar{1}011\}$ is accomplished via exchanging mechanism with kinetic barriers of 0.06~0.10 eV, and along $\langle 11\bar{2}0\rangle$ steps via hopping mechanism with kinetic barriers of 0.14~0.19 eV. The three-dimensional Ehrlich–Schwoebel barrier is converged as the step height exceeds three atomic layers.

## Methods

First principles density function theory calculations were conducted using VASP.5.2. We used generalized gradient approximation (GGA) for the exchange correlation functional with the Perdew-Becke-Erzenhof (PBE)^46, 47^ parameterization, and *projector augmented wave* (PAW) pseudopotentials for the interaction between valence electrons and ionic cores^48^. The number of valence electrons in the pseudo-potentials are 2 (3 s^2^) for Mg^49^. We used a plane wave cutoff of 500 eV and 19 × 19 × 11 Γ-centered Monkhorst Pack k-point mesh for the integration of primitive hexagonal Brillouin zone (BZ)^50^. An optimized structure was obtained when the force on each atom is smaller than 0.0001 eV/nm. The optimized parameters for hcp-Mg are a = 0.3192 nm and c = 0.5178 nm for the lattice parameters, giving c/*a* = 1.622, and −1.516 eV for the cohesive energy. To compute surface energies, as shown in Fig. S1, periodic boundaries are adopted for both the *x*-axis ($[10\bar{1}2]$) and the *z*-axis ($[1\bar{2}10]$), and two free surfaces along the *y*-axis ($[\bar{1}011]$) is achieved by adding additional vacuum space. The dimensions in the *x* and *z* directions correspond to one unit length. The dimension in the *y* direction varies in order to eliminate the size effect. The same calculations are also performed for Mg with two existing empirical potentials, one developed by Liu *et al*. using forcing matching method (referred to as Liu-Mg)^43^, and the recent one developed by Sun *et al*. in the embedded atom method (EAM) potential (referred to as Sun-Mg)^44^. The same boundary conditions as that in DFT are adopted in MS calculations. The dimensions are much bigger than that in DFT, not less than 5 nm in both *x* and *z* directions. The relaxation is accomplished by molecular dynamics (MD) method at 1 K for 10 ps, and following dynamics quenching until the max force acting on each atom less than 5 pN. In the calculations of surface clusters, one of the two free surfaces along the *y*-axis is fixed. The energetic barriers corresponding to surface diffusion of adatom and surface clusters are calculated by using NEB method, searching for minimum energy path (MEP)^45^.

## Electronic supplementary material

Supplementary
