# Supplementary material for: Clustering on Magnesium Surfaces – Formation and Diffusion Energies
Source: Sci Rep. 2017 Jul 12;7:5167. doi: 10.1038/s41598-017-05366-1 (PMC5507928; doi:10.1038/s41598-017-05366-1)
Supplement: Supplementary file 1 — Supplementary [file 41598_2017_5366_MOESM1_ESM.pdf]

Supplementary

Clustering on Magnesium Surfaces – Formation and Diffusion Energies

Haijian Chu[1,2,*], Hanchen Huang[3], Jian Wang[4,*]

[1] Department of Mechanics, Shanghai University, Shanghai 200444, China

[2] Shanghai Institute of Applied Mathematics and Mechanics, Shanghai University, Shanghai 200444, China

[3] Department of Mechanical and Industrial Engineering, Northeastern University, Boston, MA 02115, USA

[4] Department of Mechanical and Materials Engineering, University of Nebraska-Lincoln, Lincoln, NE 68588, USA

Supplementary information

S1. Surface energy (Figures S1-S2)

S2. Adatom diffusion on surface {0001}

S3. Multiple configurations of a cluster on surface (0001) (Figure S3)

S4. Cluster diffusion on surface (0001) (Figures S4-6)

S5. Adatom diffusion on surface {$\bar{1}$011}

S6. DFT and MS calculations for surface steps (Figure S7)

S7. Adatom diffusion along and down steps with the facet ($\bar{1}$101) (Figures S8-S10)

S8. Adatom diffusion along and down steps with the facet (1$\bar{1}$01) (Figures S11-S13)

S9. Adatom diffusion along and down steps on surface ($\bar{1}$101) (Figures S14-S16)

Supplementary

S1. Surface energy

To compute surface energies, periodic boundaries are adopted for both the *x*-axis ($[10\bar{1}2]$) and the *z*-axis ($[1\bar{2}10]$), and two free surfaces along the *y*-axis ($[\bar{1}011]$) is achieved by adding additional vacuum space. The dimensions in the *x* and *z* directions correspond to one unit length. The dimension in the *y* direction varies in order to eliminate the size effect. The dimensions of MS simulation cells are much bigger than that in DFT, not less than 5 nm in both *x* and *z* directions. The relaxation is accomplished by molecular dynamics (MD) method at 1 K for 10 ps, and following dynamics quenching until the max force acting on each atom less than 5 pN. In the calculations of surface clusters, one of the two free surfaces along the *y*-axis is fixed.

Figure S1 shows the simulation cell for surface $\{\bar{1}011\}$ in MS simulations. Crystallographic plane $\{\bar{1}011\}$ includes two atoms in a unit cell (defined by the parallelogram), but they are in different heights and form a rumpled plane including *A* and *B* planes (Fig. S1b). In MS calculation, the dimensions in both the *x*-axis and *z*-axis are greater than 2.5 nm. The dimension in the *y*-axis varies from 5 nm to 15 nm in order to eliminate the size effect on surface energy. In DFT calculation, the dimensions in the *x*-*z* plane are the same as the unit cell marked in Fig. S1c, and the dimension in the *y*-axis varies in the range of 2 nm ~ 10 nm in order to eliminate the size effect. 9x1x9 Γ-centered Monkhorst Pack k-point mesh with respect to the *x*-, *y*-, and *z*-axis is used in these calculations. In principle, if the size effect can be eliminated, the total potential energy of the model can be written as

$$E_{(N)} = NE_{coh} + 2AE_s \quad (1)$$

Where N is the number of atoms in the simulation cell, $E_{(N)}$ is the total potential energy of the simulation system, E_{coh} is the cohesive energy of Mg atom in a perfect single crystal, E_s is the surface formation energy, and A is the area of the surface. Thus, the surface formation energy is

$$E_s = \frac{E_{(N)} - NE_{coh}}{2A} \quad (2)$$

The variation of surface energies with the size of simulation cell for four most compact crystallographic planes indicates that surface energies reach a constant value as the

thickness is greater than 7 nm, as shown in Figure S2. In addition, numerical results show that, except of (0001), the surface energy of the surface with a single-atom terminated plane (terminated by plane A or B as shown in Fig. S1b) is always larger than that with double-atoms terminated plane (A and B together).

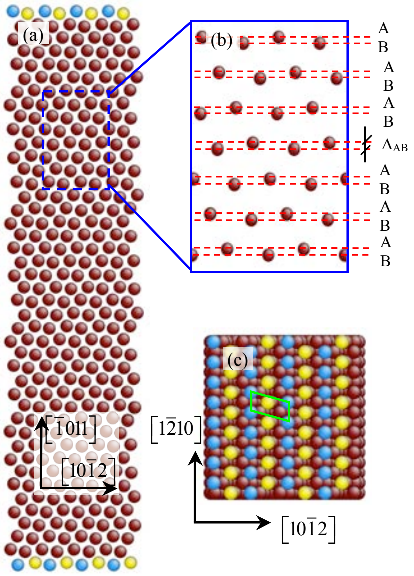

Figure S1. (a) The simulation cell for surface $\{\bar{1}011\}$ in molecular statics simulations. The periodic boundaries are adopted in the *x*-axis along $[10\bar{1}2]$ and *z*-axis along $[1\bar{2}10]$. The *y*-axis is along $[\bar{1}011]$. (b) Magnification of the crystallographic plane, showing a rumpled plane including plane A and plane B. (c) The projection of surface $\{\bar{1}011\}$ on the y-direction. A boundary unit cell in DFT calculation is defined by the parallelogram.

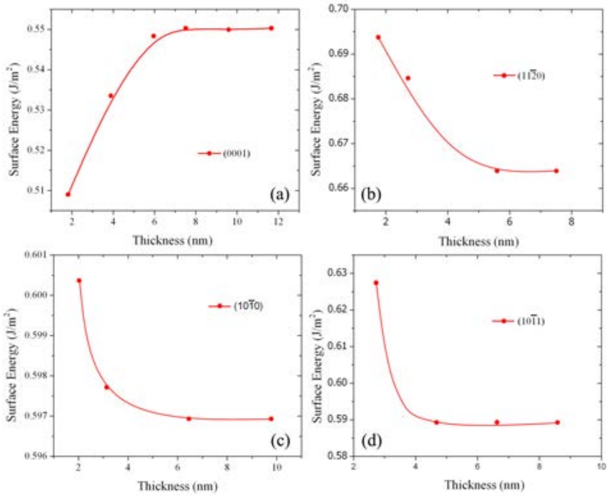

Figure S2. The variation of surface energies obtained by DFT as a function of the dimension along the *y*-axis with respect to different surfaces, (a) (0001), (b) $(11\bar{2}0)$, (c) $(10\bar{1}0)$, and (d) $(10\bar{1}1)$.

S2. Surface {0001}

Surface (0001) have the similar feature to fcc {111}. We performed a systematical study for surface clusters with both DFT and the empirical potential Liu-Mg. The simulation models consist of 14401 atoms in MS simulations and 281 atoms in DFT calculations. The periodic boundary is adopted in the *x*-*z* plane, the fixed boundary is adopted in the bottom region of the simulation cell (y-axis) and the free surface in the top along the *y*-axis. The dimensions of MS simulation cell are 8.3 nm in the *x*-axis, 6.4 nm in the *z*-axis, and 6.2 nm in the *y*-axis. The fixed region in height is two times of the cutoff distance of the Liu-Mg potential. The dimensions of the simulation cell in DFT calculation are 2.2 nm, 1.8 nm, and 1.6 nm with respect to the *x*-, *y*- and *z*-axis. Correspondingly, the calculations were done with the K-point of 4×4×4. To obtain the surface potential landscape in association with the diffusion of an adatom, we firstly obtain a fully relaxed free surface without the adatom on surface. After this, one adatom is located at different positions that are defined in a fine-meshed grid within a unit cell of {0001} surface. The distance between two neighboring grids is 0.04 nm. During MS relaxation, the adatom only has one freedom along the y-axis.

S3. Multiple configurations for a 3-atom cluster on surface (0001).

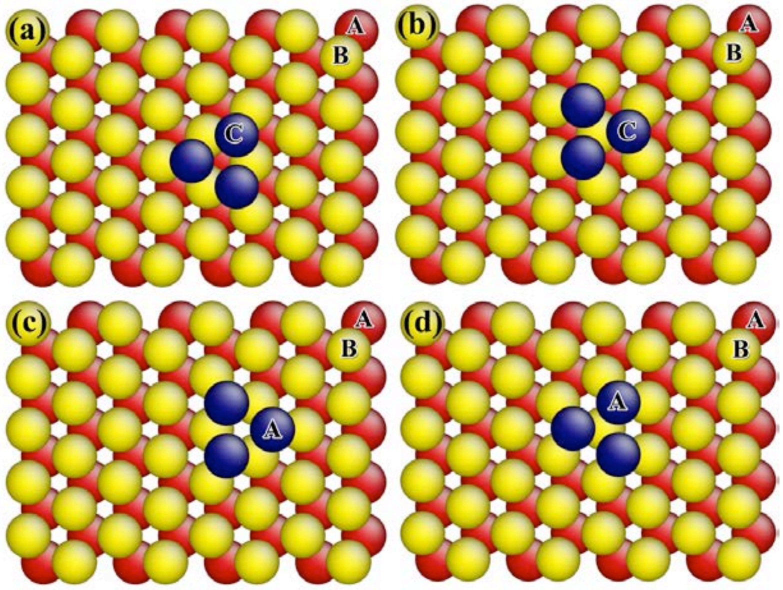

Figure S3. Atomic structures of a 3-atom cluster. (a) and (b) the cluster sites at F-sites. (c) and (d) the cluster sites at H-sites. A, B, and C refer to the stacking of fcc (111) plane.

S4. Cluster diffusion on surface (0001).

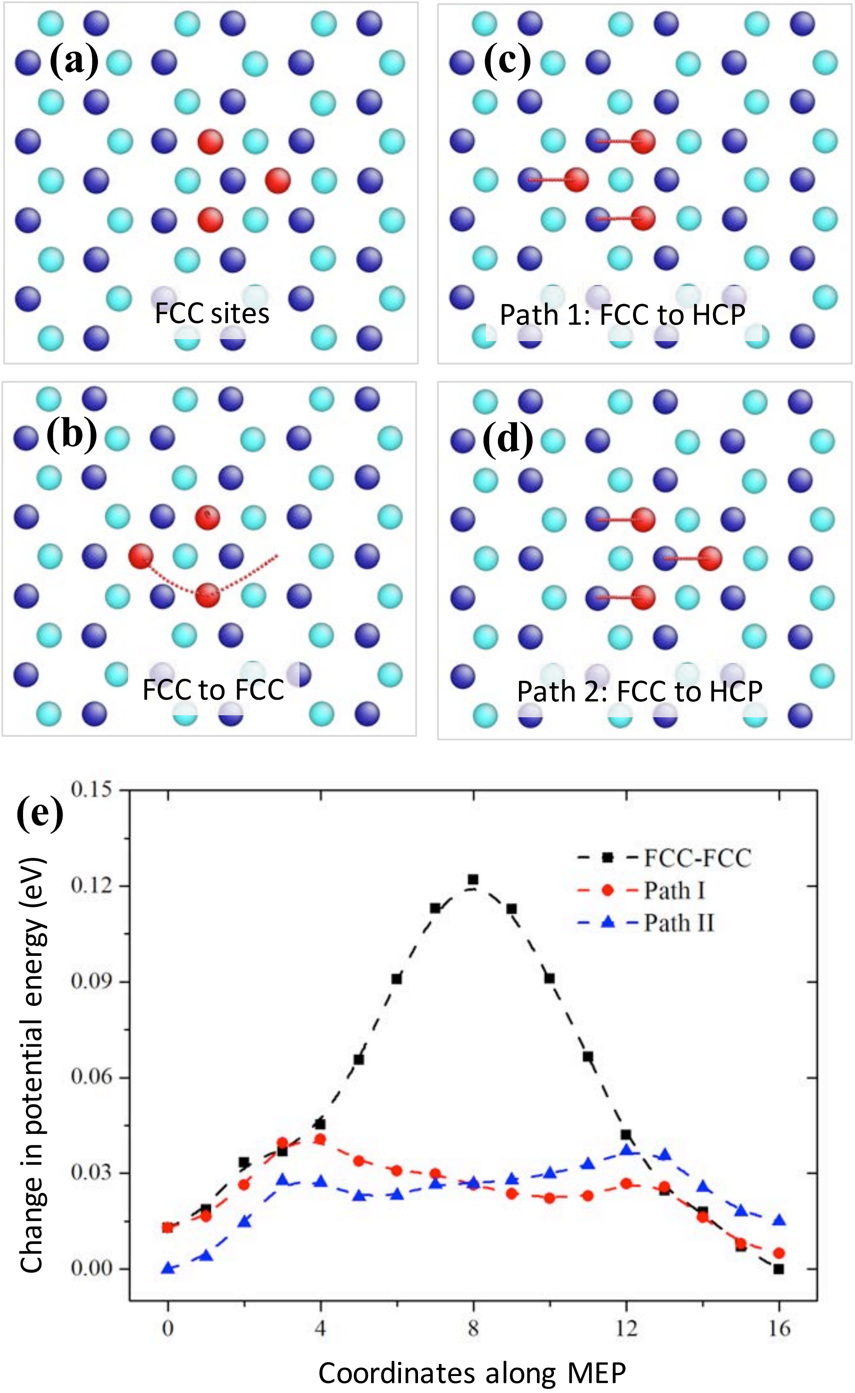

Figure S4. Atomic structures of 3-atom clusters, showing (a) 3 atoms at fcc sites, (b) the transition from one set of fcc sites to another set of fcc sites, (c) the transition from one set of fcc sites to one set of hcp sites, and (d) the transition from one set of fcc sites to another set of hcp sites. (e) The variation of potential energy with the coordinate along the minimum energy paths.

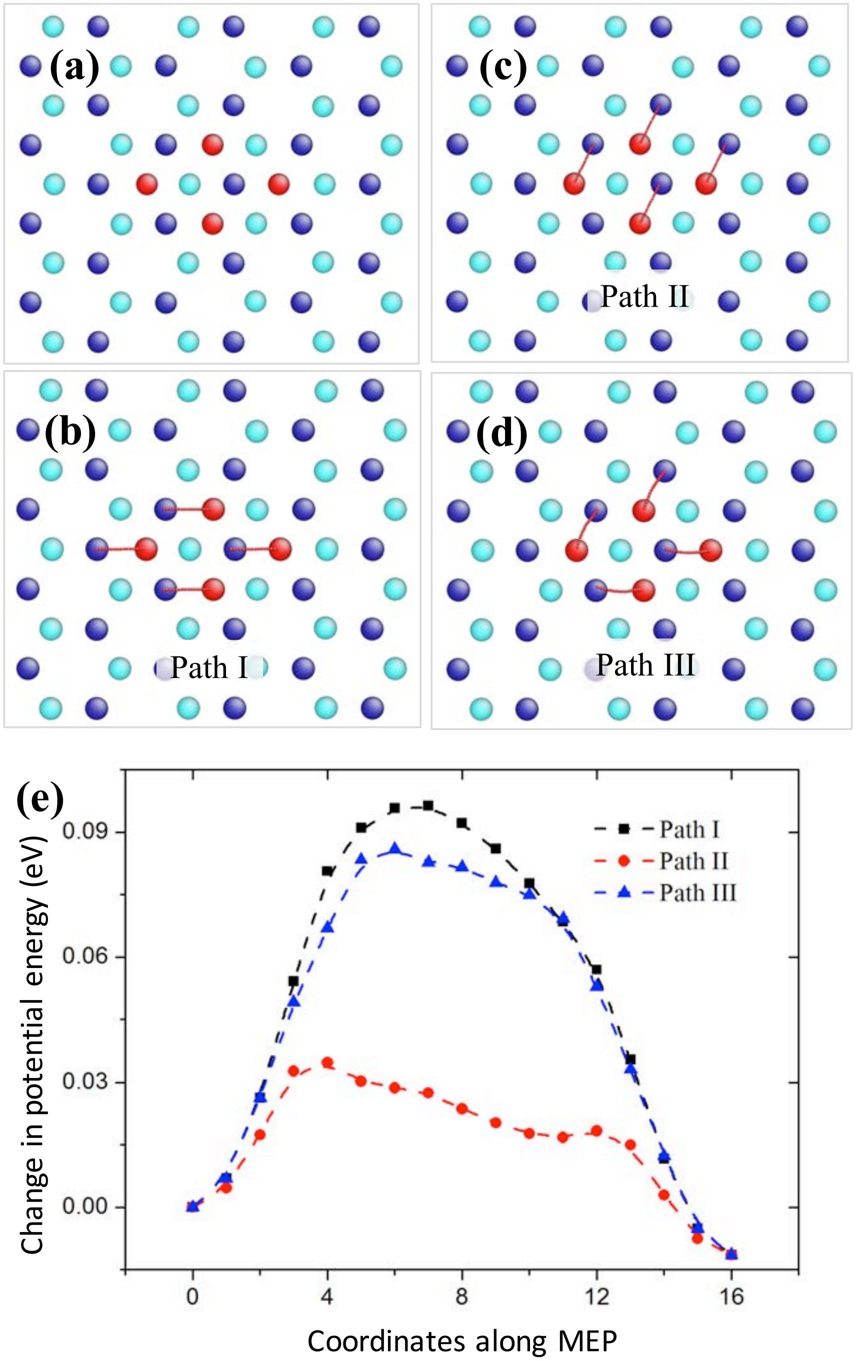

Figure S5. Atomic structures of 4-atom clusters, showing (a) 4 atoms at fcc sites, (b)-(d) the transitions from fcc sites to hcp sites through three paths. (e) The variation of potential energy with the coordinate along the minimum energy paths.

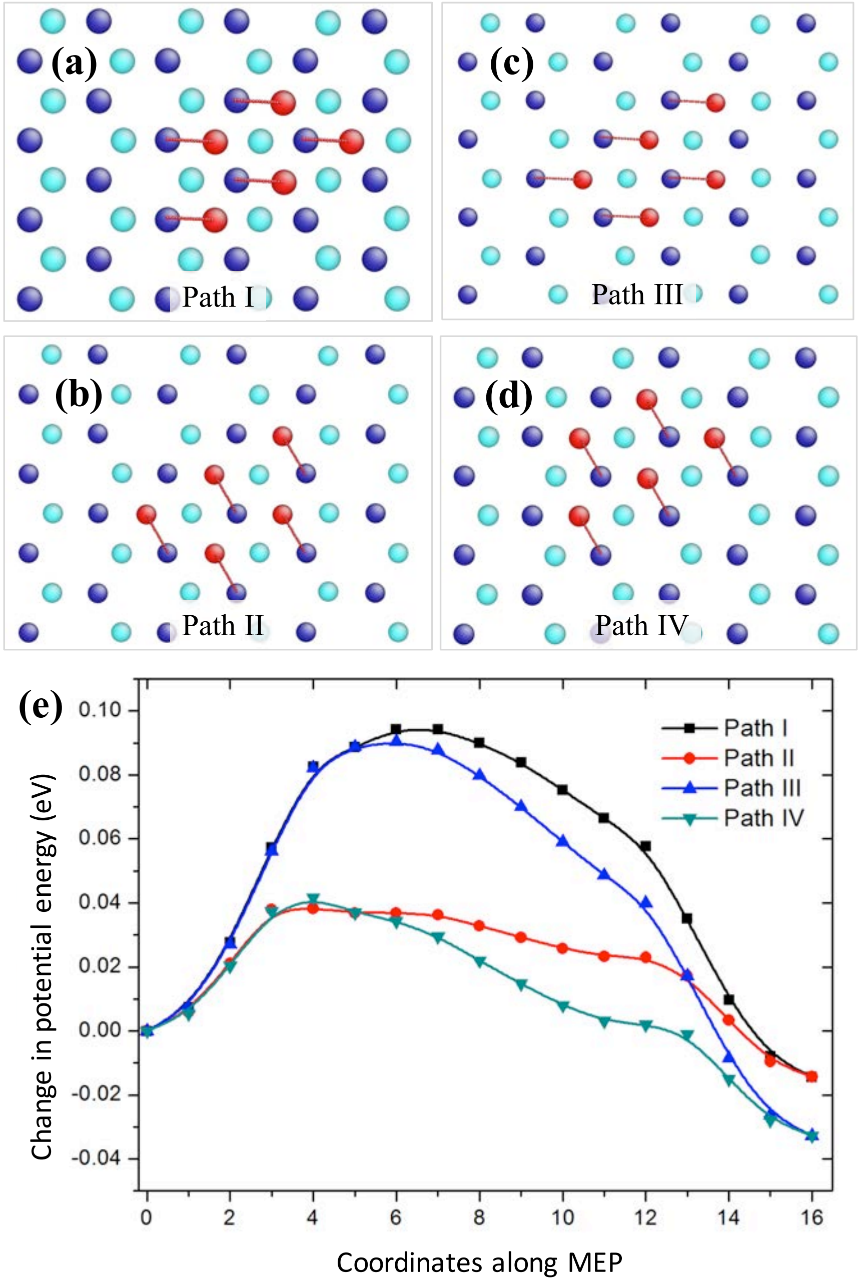

Figure S6. Atomic structures of 5-atom clusters, showing (a)-(d) the transition from fcc sites to hcp sites through four paths. (e) The variation of potential energy with the coordinate along the minimum energy paths.

S5. Surface $\{\bar{1}011\}$

Surface $\{\bar{1}011\}$ is a rumpled crystallographic plane, which could be terminated by plane A or plane B. We performed a systematical study for surface clusters with both DFT and MS with the empirical potential Liu-Mg. The model consists of 14081 atoms in MS simulation and 211 atoms in DFT calculation. The periodic boundary is adopted in the *x*-*z* plane, with the *x*-axis along $[10\bar{1}2]$ and the *z*-axis along $[1\bar{2}10]$. The *y*-axis is normal to $(\bar{1}011)$ plane and the fixed boundary is adopted in the low region of simulation cell. The dimensions of MD simulation cell are 4.7 nm in the *x*-axis, 5.1 nm in the *z*-axis, and 14.2 nm in the y-axis. The fixed region is two times of cutoff distance in height. The dimensions of simulation cell in DFT calculation are 1.8 nm, 1.8 nm, and 1.6 nm in the *x*, *y* and *z* axes. The calculations were done with the K-point 4×4×4.

S6 DFT and MS calculations for surface steps

Figure S7a shows the simulation cell of surface (0001) containing two single steps in the left (a facet $(\bar{1}101)$) and the right (a facet $(1\bar{1}01)$) corresponding to the Wulff construction. The periodic boundaries are adopted in the *x*- and *z*-axis. In order to eliminate the step-step interaction, the DFT and MS calculations were conducted with different spacing *d* between the two steps. The results are plotted in Fig. S7b, suggesting the optimized simulation dimension in the x-axis, 5 nm in DFT (2.5 nm between two steps) and 10 nm in MS (5 nm between two steps). With the suggested dimensions, we calculated the energy barrier of adatom diffusion along the step by the hopping mechanism. Using NEB method with empirical potential Liu-Mg, Fig. S7c shows the variation of the relative potential energy with the coordinates along the diffusion path. In the DFT calculation, we calculated the formation energy of an adatom at several different positions along the diffusion path. For each configuration, the DFT relaxation was performed with fixing one freedom of degree of the adatom along the diffusion path and freeing other two freedoms of degree. The energy barrier is calculated corresponding to the difference between the maximum and the minimum. The results show that the energy barrier is 0.124 eV (MS) and 0.139 eV (DFT), with a relative error of 10.8%, which is reasonably accepted for atomistic simulations with empirical potential.

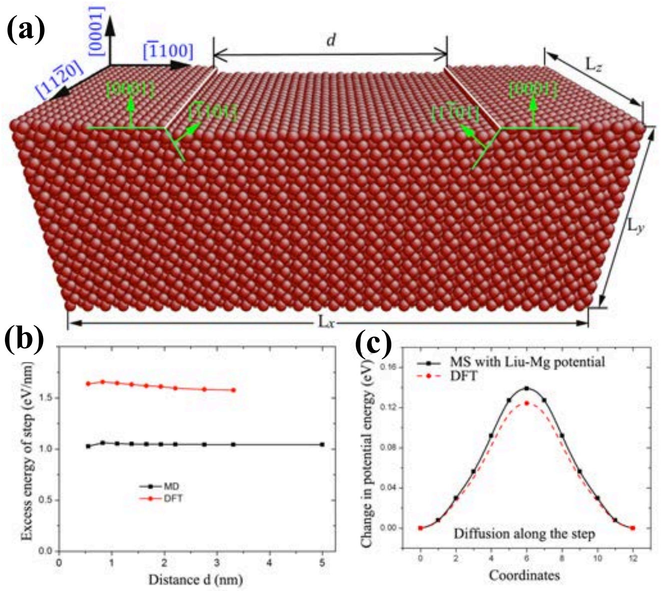

Figure S7. (a) Simulation cell of surface (0001) containing two single steps in the left (a facet ($\bar{1}101$) and the right (a facet ($1\bar{1}01$). The dimension L_x is double the distance of d. (b) Variation of step formation energy with different spacings *d* between the two steps, (c) Minimum energy path of adatom diffusion along the left step.

S7. Steps with facet ($\bar{1}101$) on surface (0001):

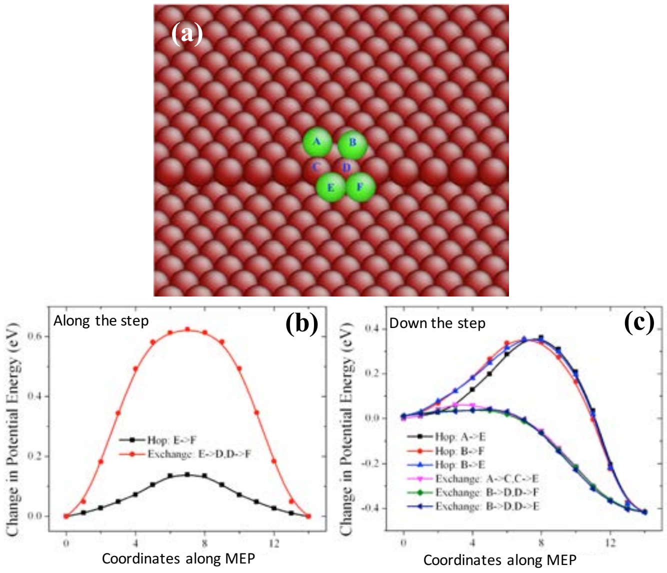

Figure S8. (a) Diffusion paths for adatom in the left step corresponding to the ledge between (0001) and ($\bar{1}101$), (b) Minimum energy paths of adatom diffusion along the left step by exchanging and hopping mechanisms, (c) Minimum energy paths of adatom diffusion down the step by exchanging and hopping mechanisms. Green atoms represent the stable locations for adatom.

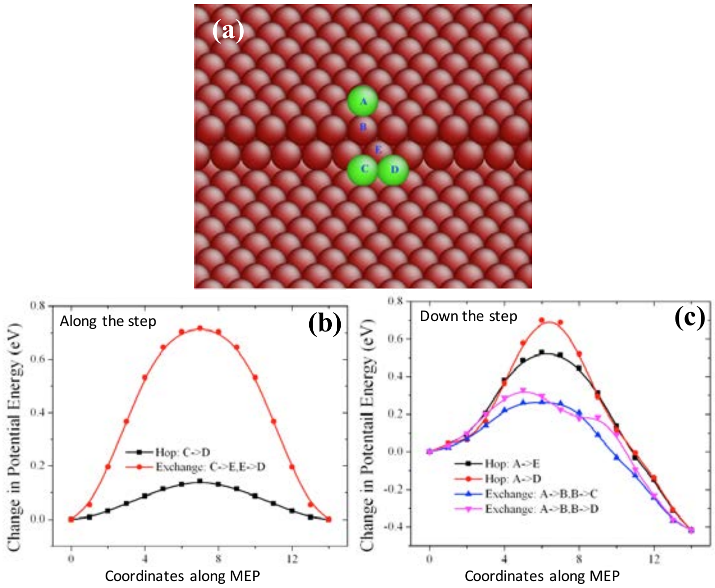

Figure S9. (a) Diffusion paths for adatom in the left two-layer step corresponding to the ledge between (0001) and ($\bar{1}101$), (b) Minimum energy paths of adatom diffusion along the left step by exchanging and hopping mechanisms, (c) Minimum energy paths of adatom diffusion down the step by exchanging and hopping mechanisms. Green atoms represent the stable locations for adatom.

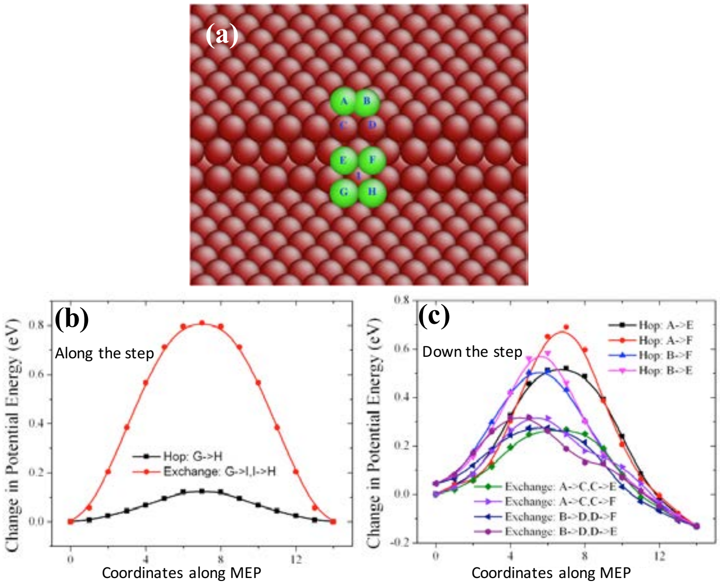

Figure S10. (a) Diffusion paths for adatom in the left three-layer step corresponding to the ledge between (0001) and ($\bar{1}101$), (b) Minimum energy paths of adatom diffusion along the left step by exchanging and hopping mechanisms, (c) Minimum energy paths of adatom diffusion down the step by exchanging and hopping mechanisms. Green atoms represent the stable locations for adatom.

S8. Steps with facet (1$\bar{1}$01) on surface (0001):

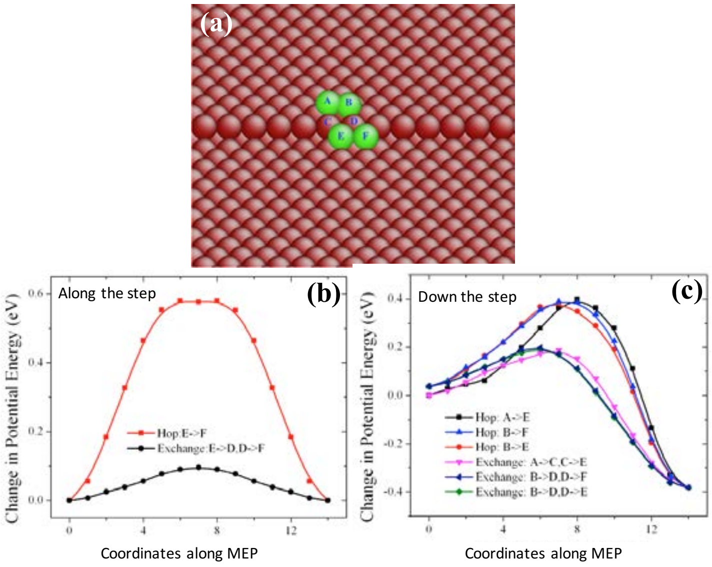

Figure S11. (a) Diffusion paths for adatom in the right step corresponding to the ledge between (0001) and (1$\bar{1}$01), (b) Minimum energy path of adatom diffusion along the right step by exchanging and hopping mechanisms, (c) Minimum energy path of adatom diffusion down the right step by exchanging and hopping mechanisms. Green atoms represent the stable locations for adatom.

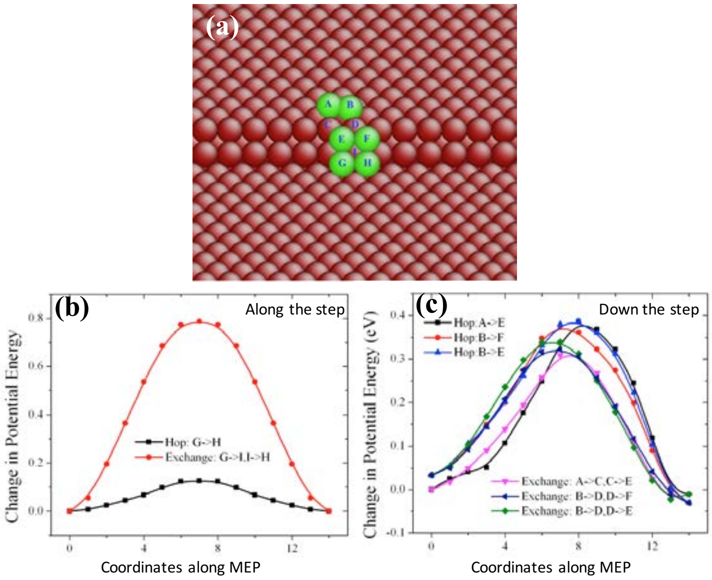

Figure S12. (a) Diffusion paths for adatom in the right 2-layer step corresponding to the ledge between (0001) and (1$\bar{1}$01), (b) Minimum energy path of adatom diffusion along the right step by exchanging and hopping mechanisms, (c) Minimum energy path of adatom diffusion down the right step by exchanging and hopping mechanisms. Green atoms represent the stable locations for adatom.

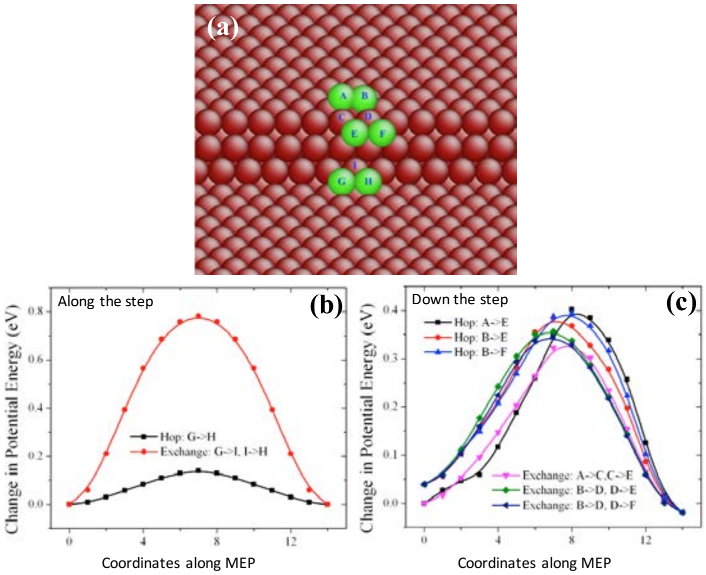

Figure S13. (a) Diffusion paths for adatom in the right 3-layer step corresponding to the ledge between (0001) and ($1\bar{1}01$), (b) Minimum energy path of adatom diffusion along the right step by exchanging and hopping mechanisms, (c) Minimum energy path of adatom diffusion down the right step by exchanging and hopping mechanisms. Green atoms represent the stable locations for adatom.

S9. Steps on surface ($\bar{1}011$)

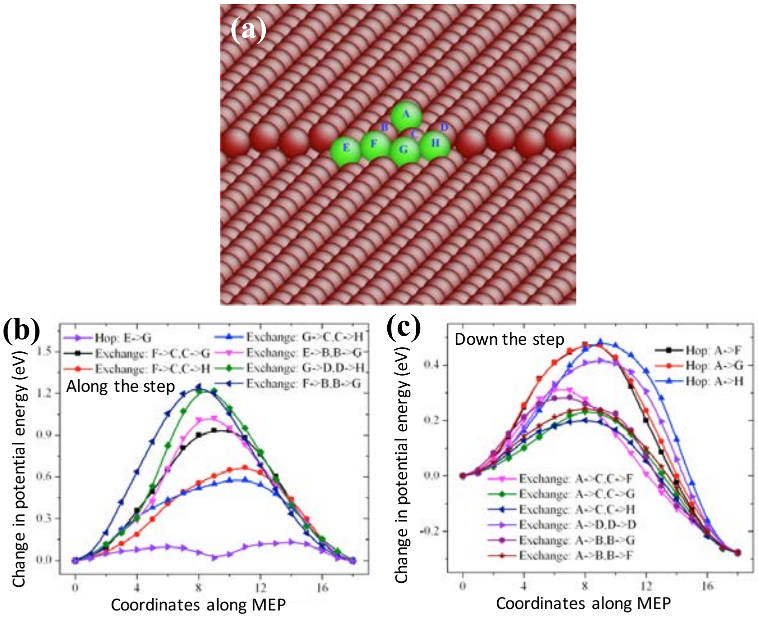

Figure S14. (a) Diffusion paths for adatom diffusion on down a one-layer step on ($\bar{1}011$), (b) Minimum energy path of adatom diffusion along the step by exchanging and hopping mechanisms, (c) Minimum energy path of adatom diffusion down the step by exchanging and hopping mechanisms. Green atoms represent the stable locations for adatom.

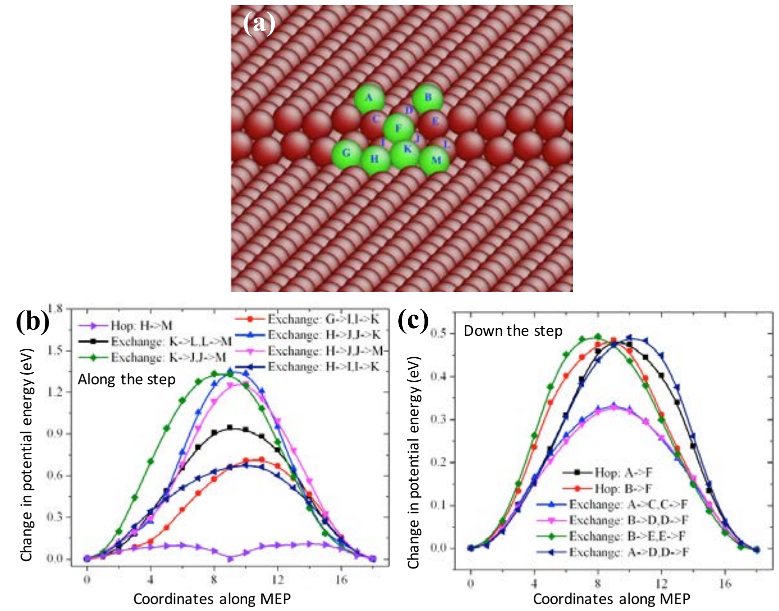

Figure S15. (a) Diffusion paths for adatom diffusion down a two-layer step on $(\bar{1}011)$, (b) Minimum energy path of adatom diffusion along the step by exchanging and hopping mechanisms, (c) Minimum energy path of adatom diffusion down the step by exchanging and hopping mechanisms. Green atoms represent the stable locations for adatom.

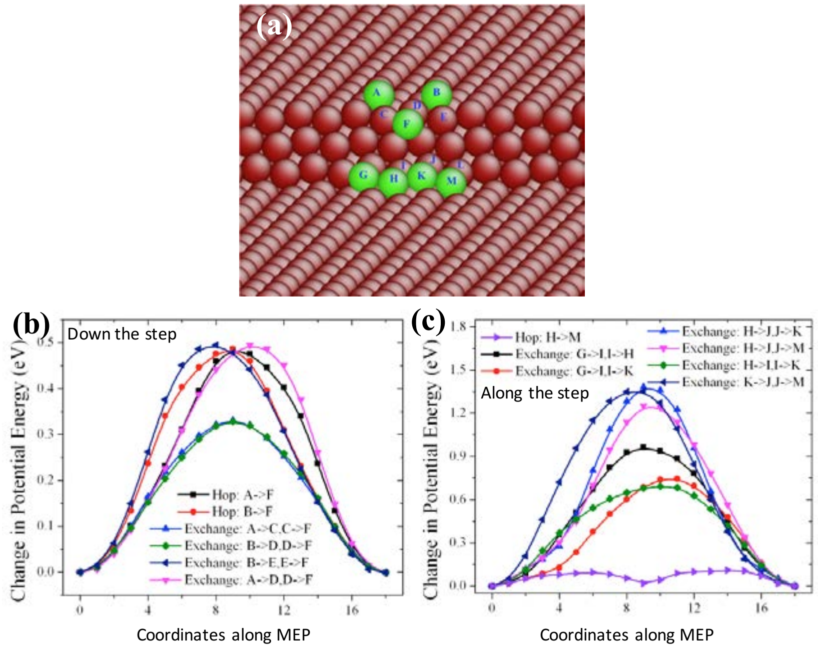

Figure S16. (a) Diffusion paths for adatom diffusion down a three-layer step on $(\bar{1}011)$, (b) Minimum energy path of adatom diffusion down the step by exchanging and hopping mechanisms, and (c) Minimum energy path of adatom diffusion along the step by exchanging and hopping mechanisms. Green atoms represent the stable locations for adatom.
